# Clusterin induced by N,N′-Dinitrosopiperazine is involved in nasopharyngeal carcinoma metastasis

**DOI:** 10.18632/oncotarget.6750

**Published:** 2015-12-24

**Authors:** Yuejin Li, Jinping Lu, Shan Zhou, Weiwei Wang, Gongjun Tan, Zhenlin Zhang, Zigang Dong, Tiebang Kang, Faqing Tang

**Affiliations:** ^1^ State Key Laboratory of Oncology in South China and Department of Experimental Research, Sun Yat-Sen University Cancer Center, Guangzhou 510060, Guangdong, China; ^2^ Clinical Laboratory and Medical Research Center, Zhuhai Hospital, Jinan University, Zhuhai People's Hospital, Zhuhai 519000, Guangdong, China; ^3^ Hormel Institute, University of Minnesota, Austin, Minnesota 55912, USA

**Keywords:** N, N′-Dinitrosopiperazine, clusterin, nasopharyngeal carcinoma, metastasis

## Abstract

Nasopharyngeal carcinoma (NPC) has a high metastatic clinicopathological feature. As a carcinogen factor, N,N′-Dinitrosopiperazine (DNP) is involved in NPC metastasis, but its precise mechanism has not been fully elucidated. Herein, we showed that DNP promotes NPC metastasis through up-regulating anterior clusterin (CLU). DNP was found to increase CLU, matrix metalloproteinases (MMP) 9 and vascular endothelial growth factor (VEGF) expression and activity, further DNP-increased MMP-9 and VEGF expression was through up-regulating CLU. We also found that DNP increased the binding of CLU with MMP-9 or VEGF. DNP induced the motility and invasion of NPC cell, which was inhibited by siRNA-CLU. The clinical investigation showed that CLU, MMP-9 and VEGF were positively correlated with the tumor-node -metastasis (TNM) classification. These results indicate that DNP may promote NPC tumor metastasis through up-regulating CLU, MMP-9 and VEGF expression. Therefore, DNP-increased CLU expression may be an important factor of NPC-high metastasis, and CLU may serve as a biomarker for NPC metastasis.

## INTRODUCTION

Nasopharyngeal carcinoma (NPC) is common malignant cancer in southern China [1]. Epidemiological investigations revealed that incidence of NPC has remained high in endemic regions, particularly in southeast Asia with an incidence of 30-80 per 100,000 people per year in southern China [2, 3]. In spite of significant advancement in early diagnosis, surgical intervention as well as local and systemic adjuvant therapies, the majority of cancer deaths are attributable to tumor invasion and distant metastasis that are resistant to available therapies [4-6].

In endemic NPC, > 95% is classified as the undifferentiated World Health Organization (WHO) type III and is universally associated with Epstein–Barr virus (EBV) [7, 8] and dietary intake of preserved foods [9]. Moreover, in studies on Chinese populations in high incidence regions, the relative risk of NPC is related to their eating habits of the region, especially with dietary intake of salt-preserved fish [9-16]. The process of salt preservation is inefficient and become partially putrefied, consequently, these foods accumulate significant levels of nitrosamines [17, 18], which are known carcinogens [17, 19, 20]. N,N′-Dinitrosopiperazine (DNP) is one predominant volatile nitrosamine in salted fish [14, 21]. The carcinogenic potential of DNP in salt-preserved fish is supported by experiments in rats, which develop malignant nasal and NPC [22-24]. Furthermore, DNP can induce malignant transformation of human embryonic nasopharyngeal epithelial cells [25].

Our previous works have shown that DNP induces NPC and shows organ specificity to the nasopharyngeal epithelium, and is involved in not only nasopharyngeal tumorigenesis but also metastasis [26, 27]. Withal, a quantitative proteomic study using the stable isotope labeling with amino acids in cell culture (SILAC) coupled with mass spectrometry was used to investigate biomolecules of DNP-induced NPC metastasis. And some metastasis adhesion proteins were found a significant increase in NPC metastasis, such as clusterin (CLU), matrix metallo-proteinases (MMP), vascular endothelial growth factor (VEGF) and etc [28, 29], but its regulatory mechanism is not clear.

CLU is a protein widely distributed among cells. It is apparently involved in many biological processes such as tissue differentiation, cell adhesion, cell–cell or cell–substratum interaction. Recently, it was found that CLU is involved in many pathological states such as neurodegeneration and cancer [30, 31]. CLU is up-regulated in various cancers [32-34], further studies showed that CLU is regulated by oncogenes and oncoproteins [35]. Importantly, CLU is associated with a metastatic phenotype [36], its mechanism is to regulate MMP-2, MMP-9, VEGF and E-cadherin expression [37-40].

MMPs promote modifications of tissue extracellular matrix, and trigger the development of intravasation-sustaining neovasculature at the stages of tumor growth and progression [41-44]. VEGF is an angiogenic cytokine expressed by tumors and a vascular permeability factor, which being considered a key regulator in tumor-induced neoangiogenesis [45-48]. Furthermore, some studies suggest that CLU may regulate aggressive behavior of human clear renal cell cancer (CRCC), ovarian cancer, and colon cancer cells through MMP-9 or VEGF expression [38-40, 49]. In the current study, we explored the role and possible mechanism of CLU in NPC metastasis induced by DNP. Our results showed that DNP promoted NPC metastasis through up-regulating CLU. DNP was found to induce the motility and invasion of NPC cell, and this induction was inhibited by siRNA-CLU. We also found that DNP increased MMP-9 and VEGF expression through up-regulating CLU expression. The clinical data showed that CLU was positively correlated with the tumor-node-metastasis (TNM) classification. Therefore, DNP-induced CLU expression may be an important factor of NPC-high metastasis.

## RESULTS

### CLU, MMP-9 and VEGF expressions and its clinical significance in NPC

**Figure 1:**
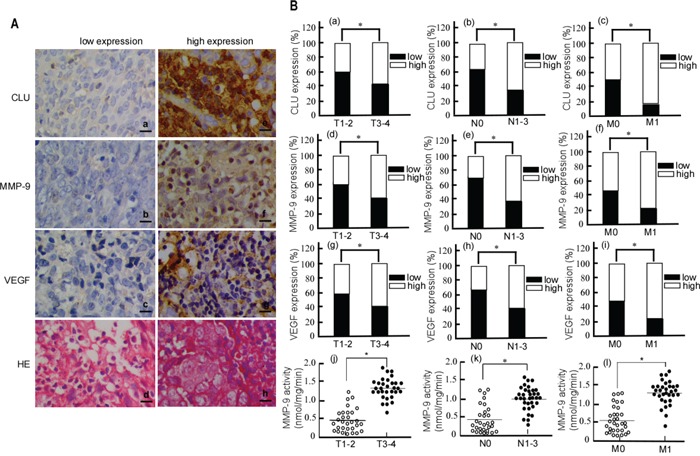
The immunohistochemical staining of CLU, MMP-9 and VEGF in NPC tissues **A.** Representative images of CLU, MMP-9 and VEGF protein expression in paraffin-embedded tissue from patients with NPC. CLU, clusterin; MMP-9, matrix metalloproteinases 9; VEGF, vascular endothelial growth factor; HE, hematoxylin and eosin. Original magnification, ×400. Scale bar, 5μm. **B.** Graphical illustration of statistical CLU, MMP-9 and VEGF distribution in NPC tissues. T, original tumor size and nearby tissue invasion; N, lymph node metastasis; M, distant metastasis. *p < 0.05.

To clarify the relationship of NPC and CLU, MMP-9 or VEGF expression, we detected CLU, MMP-9 and VEGF expression in NPC biopsy tissues using immunohistochemistry, and analyzed their clinical significance. As shown in Figure 1A, CLU, MMP-9 and VEGF positive signals showed brown-yellow granules, there exited low (Fig.1A-a, b, c) and high (Fig. 1A-e, f, g) expression. CLU was localized in the cytoplasm and nucleus (Fig. 1A-e), MMP-9 in the cytoplasm and nucleus (Fig. 1A-f), and VEGF in the cytoplasm (Fig. 1A-g). Furthermore, the correlation of CLU, MMP-9 or VEGF expression with clinicopathological features was shown in Table 1. CLU expression was positively correlated with T stage (original tumor size and nearby tissue invasion) (Fig.1B-a. *P* < 0.05), N stage (lymph node metastasis) (Fig.1B-b. *P* < 0.05) and M stage (distant metastasis) (Fig.1B-c. *P* < 0.05). MMP-9 was also positively correlated with T stage (Fig.1B-d. *P*< 0.05), N stage (Fig.1B-e. *P* < 0.05) and M stage (Fig.1B-f. *P* < 0.05). VEGF was positively associated with T stage (Fig.1B-g. *P* < 0.05), N stage (Fig.1B-h. *P* < 0.05) and M stage (Fig.1B-i. *P* < 0.05). Since MMP-9 protein expression may not represent its activity, we further detected MMP-9 activity in NPC biopsy sample. The result showed that MMP-9 activity at T3-4 was higher than that at T1-2 (Fig.1B-j. *P* < 0.05), N1-3 higher than N0 (Fig.1B-k. *P* < 0.05), and M1 higher than M0 (Fig.1B-l. *P* < 0.05). MMP-9 activity was also positively correlated with TNM stage. But all of CLU, MMP-9 and VEGF expressions did not link with age, or gender (Table 1). These results indicated that dysregulation of CLU, MMP-9 and VEGF might be related to NPC development and metastasis.

**Table 1:** CLU, MMP-9 and VEGF expression in NPC and the correlation with clinical features

Characteristic	Cases	CLU expression		*P*	MMP-9 expression		*P*	VEGF expression		*P*
		low	high		low	high		low	high	
All patients									
Gender										
Male	108	51(47.2%)	57(52.8%)	1.000	53(49.1%)	55(50.9%)	0.923	50(46.3%)	58(53.7%)	0.847
Female	36	17(47.2%)	19(52.8%)		18(50%)	18(50%)		16(44.4%)	20(55.6%)	
Age (yrs)										
< 50	80	39(48.8%)	41(51.3%)	0.970	35(43.8%)	45(56.3%)	0.351	34(42.5%)	46(57.5%)	0.600
≥ 50	64	31(48.4%)	33(51.6%)		33(51.6%)	31(48.4%)		30(46.9%)	34(53.1%)	
T stage										
T1-T2	60	37(61.7%)	23(38.3%)	0.012	38(63.3%)	22(36.7%)	0.015	35(58.3%)	25(41.7%)	0.049
T3-T4	84	34(40.5%)	50(59.5%)		36(42.9%)	48(57.1%)		35(41.7%)	49(58.3%)	
N stage										
N0	60	39(65%)	21(35%)	0.000	42(70%)	18(30%)	0.000	40(66.7%)	20(33.3%)	0.003
N1-N3	84	29(34.5%)	55(65.5%)		31(36.9%)	53(63.1%)		35(41.7%)	49(58.3%)	
M stage										
M0	126	65(51.6%)	61(48.4%)	0.006	60(47.6%)	66(52.4%)	0.043	61(48.4%)	65(51.6%)	0.037
M1	18	3(16.7%)	15(83.3%)		4(22.2%)	14(77.8%)		4(22.2%)	14(77.8%)	

### DNP induces the expressions of CLU, MMP-9 and VEGF

**Figure 2:**
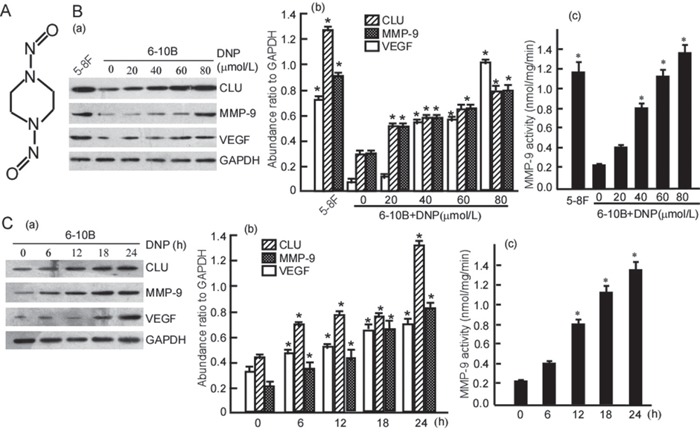
DNP induces expressions of CLU, MMP-9 and VEGF **A.** Structure of DNP, an N-nitroso compound. **B.** 6-10B cells were treated with the indicated concentration of DNP for 24 h. **C.** 6-10B cells were treated with 80 μmol/L DNP for the indicated time. CLU, MMP-9 and VEGF expressions in the DNP-treated cells were detected using Western-blotting (a). Three independent experiments were carried out, abundance ratio to GAPDH was counted, and data are represented as mean ±S.D. from three experiments (b). MMP-9 activity was measured using Fluorescent assay (c). DNP, N,N′-Dinitrosopiperazine; CLU, clusterin; MMP-9, matrix metalloproteinases 9; VEGF, vascular endothelial growth factor. *p < 0.05.

The above results showed that high expressions of CLU, MMP-9 and VEGF are associated with NPC progression. The next step is to search for the reasons of CLU, MMP-9 and VEGF high expression. As a chemical carcinogen for NPC, DNP was used in this study (Fig. 2A). To confirm whether DNP can induce CLU, MMP-9 and VEGF expression, 6-10B cells were treated with 0-80 μmol/L (non cytotoxic concentration of DNP to 6-10B was 0-100 μmol/L, shown in Supplementary Fig. 1) DNP for 24h, or with 80μmol/L DNP for 0-24 h, and then CLU, MMP-9 and VEGF expression were detected using Western-blotting. The results showed that 6-10B cells had a low expression of CLU, MMP-9 and VEGF, after DNP treatment CLU, MMP-9 and VEGF expressions dramatically increased (Fig. 2B, C. *P* < 0.05), and displayed a dose- (Fig. 2B-a, b. *P* < 0.05) and time-dependently (Fig. 2C-a, b. *P* < 0.05) manner. These findings indicated that DNP could induce CLU, MMP-9 and VEGF expression. MMP-9 activities were also detected in 6-10B cells with DNP treatment. After DNP treatment, MMP-9 activity dramatically increased, showed at dose-dependent (Fig. 2B-c. *P* < 0.05) and time-dependent (Fig. 2C-c. *P* < 0.05). DNP could also significantly increase MMP-9 activity. 5-8F cells had a high expression of CLU, MMP-9 and VEGF (Fig. 2B-a; Fig. 2B-b. *P* < 0.05), also has a high MMP-9 activity (Fig. 2B-c. *P* < 0.05). CLU, MMP-9 and VEGF highly expressed in the high metastatic 5-8F, and lowly expressed in the lowly metastatic 6-10B, which implies that CLU, MMP-9 and VEGF expressions are associate with NPC cell metastatic ability.

### DNP induces the invasion and motility of 6-10B cells through regulating CLU

**Figure 3:**
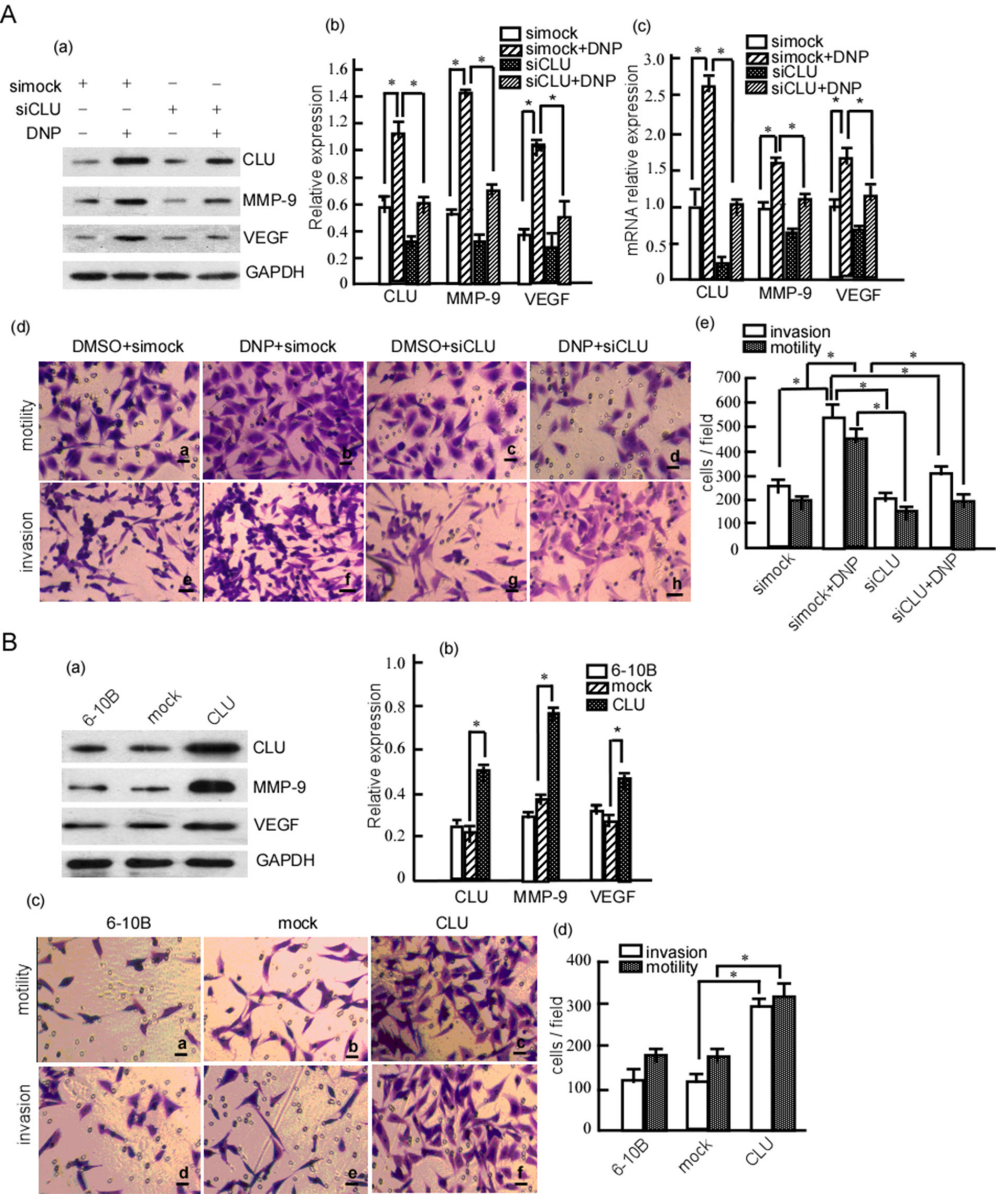
DNP-induced NPC cell invasion and motility through CLU **A.** 6-10B cells were transiently transfected with siCLU or simock, and then treated with DNP. CLU, MMP-9 and VEGF expressions were detected in the transfected cells with or without DNP treatment using Western-blotting (a), and the abundance ratios to GAPDH were calculated (b). RNA transcriptions of CLU, MMP-9 and VEGF were analyzed using qRT-PCR (c). The motility and invasion of 6-10B-siCLU and 6-10B-simock were measured using Boden chamber invasion assay (d), and the invaded cells were counted (e). **B.** 6-10B cells were transfected with pcDNA3.1 (mock) or pcDNA3.1-CLU (CLU), and the stable cell lines 6-10B-mock and 6-10B-CLU were obtained using G418 selection. CLU, MMP-9 and VEGF expression were detected in the cells using Western-blotting (a), and the abundance ratios to GAPDH were calculated (b). The motility and invasion of 6-10B-mock and 6-10B-CLU were measured using Boden chamber invasion assay (c), the invaded cells were counted (d). Data are presented as means±SD from three independent experiments. Results were analyzed by one-way ANOVA with post hoc Dunnett's test. DNP, N,N′- Dinitrosopiperazine; CLU, clusterin; MMP-9, matrix metalloproteinases; VEGF, vascular endothelial growth factor. *p < 0.05.

Besides the above, DNP induced CLU, MMP-9 and VEGF expression in NPC cells, our previous work has shown that DNP can induce NPC cell metastasis [28]. In the next step, we investigated whether DNP induces cell metastasis through CLU, MMP-9 and VEGF. 6-10B cells were transfected with siCLU, and then were treated with DNP. The results showed that the DNP-induced CLU expression was also significantly attenuated in 6-10B cells with siCLU (Fig. 3A-a, b. *P* < 0.05), MMP-9 and VEGF were also significantly suppressed in the cells with siCLU (Fig. 3A-a, b. *P* < 0.05). Simultaneously, we further confirmed whether DNP could increase *Clu*, *Mmp-9* and *Vegf* mRNA transcription using RT-qPCR. DNP could significantly increase *Clu*, *Mmp-9* and *Vegf* mRNA transcription in 6-10B cells, which were inhibited by siCLU (Fig. 3A-c. *P* < 0.05). To clarify the mechanism of DNP upregulating CLU, we used molecular dynamic simulation to calculate the interaction of DNP with CLU gene promoter, and found that DNP binds to 5′ATTG3'1 of CLU gene promoter. The various plasmids containing different CLU promoter, Clusterin–Luc plasmids, Clusterin–Luc-1998/-702, Clusterin–Luc −707/+254, Clusterin–Luc-1,998/+254, Clusterin–Luc-1,116/-702 was constructed. These plasmids were transfected into 6-10B cells, and their luciferase activities were measured. After DNP treatment, luciferase activity with clusterin–Luc-1,998/-702 and clusterin–Luc -702/+254 increased, but Clusterin–Luc-1,116/-702 did not. These findings suggest that the cis-element of CLU promoter region containing -702/t254 bp and -1,998/-1,116 bp was activated by DNP. This implies that DNP upregulates CLU through binding to cis-elements (Supplementary Fig. 2).

These cells' motility and invasion were detected. DNP-induced invasion and motility were dramatically decreased when transfected with siCLU (Fig. 3A-d, e. *P* < 0.05); however, DNP could effectively induce NPC 6-10B cell invasion and motility when CLU was not knockdown (Fig. 3A-d, e. *P* < 0.05). Further, to confirm the effect of CLU on the induction of MMP-9, VEGF expression and cell motility and invasion, 6-10B cells were transfected with pcDNA3.1 or pcDNA3.1-CLU, and then MMP-9 and VEGF expression was detected. As shown in Figure 3B, the expression of CLU, MMP-9 and VEGF were significantly up-regulated in the cells transfected with pcDNA3.1-CLU when compared with the pcDNA3.1-transfected cells and the untransfected cells (Fig. 3B-a, b. *P* < 0.05). Next, cellular invasiveness and migration were analyzed by Transwell assays, 6-10B cells transfected with pcDNA3.1-CLU exhibited a significant increase in cell motility and invasion (Fig. 3B-c, d. *P* < 0.05). Taken together, our data indicate that DNP induces MMP-9 and VEGF expression through up-regulating CLU, and promotes NPC cell invasiveness and migration.

### CLU knockdown induces significant suppression of motility and invasion in 5-8F cells

**Figure 4:**
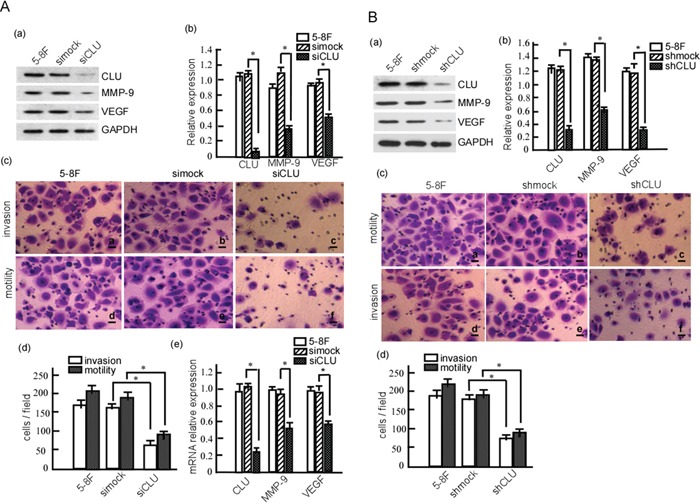
CLU knockdown reduces the invasion and metastasis of 5-8F cell **A.** 5-8F cells were transiently transfected with siCLU or simock. MMP-9 and VEGF expression were detected in these cells using Western-blotting (a), and the abundance ratios to GAPDH were calculated (b). Motility and invasion of the transfected cells were measured using Boden chamber invasion assay (c), and the invaded cells were counted (d). mRNA transcriptions of CLU, MMP-9 and VRGF were detected in the transfected cells using qRT-PCR (e). **B.** 5-8F cells were transfected with pSR-GFP/Neo -CLU-shRNA (shCLU) or pSR-GFP/Neo-NC -shRNA (shmock). 5-8F-shCLU and 5-8F-shmock stable-expressed cell lines were obtained using G418 selection. CLU, MMP-9 and VEGF expression were detected in the stable cells using Western-blotting (a), and the abundance ratios to GAPDH were calculated (b). The motility and invasion of 5-8F-shCLU and 5-8F-shmock were measured using Boden chamber invasion assay (c), and the invaded cells were counted (d). Data were from three independent experiments, expressed as means±SD. *p < 0.05. Results were analyzed by one-way ANOVA with post hoc Dunnett's test (*p < 0.05).

To confirm CLU's regulatory effect on MMP-9 and VEGR expression, 5-8F cell was transfected with siCLU, and MMP-9 and VEGR expressions were detected. We found that CLU protein was significantly attenuated (Fig. 4A-a, b. *P* < 0.05), and MMP-9 and VEGF were also significantly suppressed when CLU knockdown (Fig. 4A-a, b. *P* < 0.05). Moreover, CLU knockdown could significantly decrease *Clu, Mmp-9* and *Vegf* mRNA expression in 5-8F cells (Fig. 4A-e. *P* < 0.05; Supplementary Fig. 3). Furthermore, cell motility and invasion were detected. The data showed that motility and invasion were dramatically decreased when CLU expression was blocked (Fig. 4A-c, d. *P* < 0.05). Additionally, we also transfected pSR-GFP/Neo-NC-shRNA (shmock) or pSR-GFP/Neo-CLU-shRNA (shCLU) into 5-8F cells, and analyzed MMP-9 and VEGF expression. As shown in Figure 4B, the abundances of CLU, MMP-9 and VEGF protein were dramatically down-regulated in shCLU cells when compared with the shmock (Fig. 4B-a, b. *P* < 0.05). And the invasion and motility were significantly decreased in the cells with shCLU (Fig. 4B-c, d. *P* < 0.05). Taken together, our data show that CLU promotes the invasion and metastasis of NPC cell.

### DNP increases the binding of CLU and MMP-9 or VEGF

**Figure 5:**
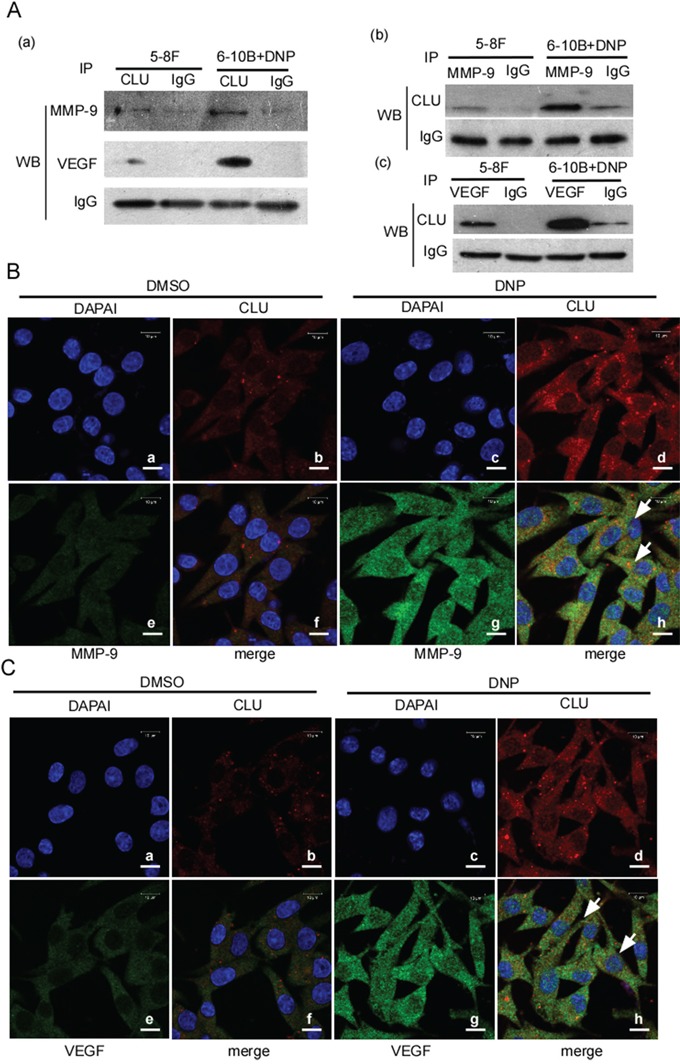
The binding of CLU with MMP-9 or VEGF **A.** the binding of CLU and MMP-9 or VEGF was determined using immunoprecipitation and Western-blotting. 5-8F cells and 6-10B cells with DNP treatment were immunoprecipitated using anti-CLU body, and MMP-9 or VEGF was detected in the immunocomplexs by Western-blotting (a). MMP-9 or VEGF was respectively immunoprecipitated in the indicated cells using MMP-9 (b) or VEGF (c) antibody, and CLU was detected using Western-blotting. IP: immunoprecipitation; WB: Western-blotting. **B.** CLU co-localizes and binds with MMP-9. **C.** CLU co-localizes and binds to VEGF. DNP-treated or untreated 6-10B cell were fixed with paraformaldehyde, stained for CLU (green) and MMP-9 or VEGF (red), and then visualized by immunofluorescence microscopy. The localization and binding of CLU and MMP-9 or VEGF were indicated. Original magnification, ×1000. Scale bar, 50 μm. Arrow, CLU binding to MMP-9 or VEGF.

The above showed that CLU highly expressed in 5-8F cells and 6-10B cells with DNP treatment conjunct MMP-9 and VEGF high expression, and knockdown CLU decreased MMP-9 and VEGF expression. The next step is to determine the binding of CLU with MMP-9 or VEGF. 5-8F and 6-10B cell with DNP treatment, which both of them have a high CLU expression, were used for immunoprecipitation with CLU antibody, and the immunocomplex was detected by Western-blotting with the MMP-9 antibody or VEGF antibody. MMP-9 and VEGF were detectable in the immunocomplex (Fig. 5A-a). Simultaneously, MMP-9 was immunoprecipitated using MMP-9 antibody in these cells, CLU was detectable in MMP-9 immunocomplex (Fig. 5A-b). And VEGF was immunoprecipitated using VEGF antibody, CLU was also detectable in VEGF immunoprecipitation (Fig. 5A-c). These results showed that CLU could bind to MMP-9 or VEGF. To further confirm whether DNP induces the binding and co-location of CLU with MMP-9 or VEGF in cell, 6-10B cells with DNP treatment or without were stained with Texas Red to detect CLU, and fluorescein isothiocyanate (FITC) for detecting VEGF by immunofluorescence. The results indicated that CLU co-localized with MMP-9 in the cytoplasm and nucleus (Fig. 5B-f, h) and VEGF in the cytoplasm and nucleus (Fig. 5C-f, h), DNP treatment increased the colocalization of CLU with MMP-9 (Fig. 5B-h) or VEGF (Fig. 5C-h).

### DNP promotes NPC cells metastasis through CLU *ex vivo*

**Figure 6:**
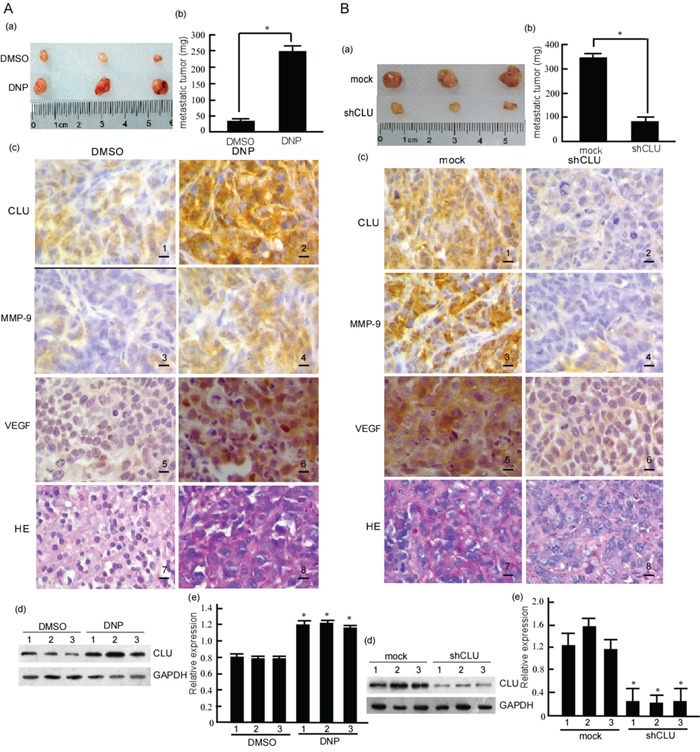
CLU's roles in DNP-induced NPC metastasis *in vivo* **A.** Nude mice were injected with 6-10B cells in Matrigel through the tail vein, and then randomly divided into two groups. One group was subcutaneously injected with DNP, the control was injected with DMSO as described in Material and Methods. The metastases to liver, lung and lymph nodes were observed (a). The metastatic tumors were weighed, the difference were analyzed (b). CLU was detected in the metastatic tumors using immunochemistry (c) and western-blotting (d), and the abundance ratios to GAPDH were calculated in the western-blotting results (e). **B.** 5-8F-shCLU and 5-8F-shmock cells in Matrigel were respectively injected in nude mice through the tail vein. The metastases to liver, lung and lymph nodes were observed (a). The metastatic tumors were weighed, the difference were analyzed (b). CLU was detected in metastatic tumors using immunochemistry (c) and western-blotting (d), and the abundance ratios to GAPDH were calculated in the western-blotting results (e). CLU, clusterin; DNP, N,N′-Dinitrosopiperazine; DMSO, Dimethyl sulfoxide; HE, hematoxylin and eosin. Original magnification, ×400. Scale bar, 5 μm. *p< 0.05.

In this study, the metastasis-inducing effects of DNP were confirmed *ex vivo*. 6-10B cells were injected into the tail veins of BABL/c mice, and then the mice were treated DNP. The metastases were observed in livers, lungs and lymph nodes of the mice. Metastases of 6-10B cells to mice livers, lungs and lymph nodes significantly increased after DNP treatment (Fig. 6A-a, b. *P* < 0.05). Simultaneously, CLU was detected in metastatic tumors by immunohistochemistry and Western-blotting. The results showed that CLU levels were high in metastatic tumors from DNP-treated mice (Fig. 6A-c, d, e. *P*<0.05). The immunohistochemistry results showed that MMP-9 and VEGF expressions were also increased in DNP treatment group (Fig. 6A-c panel 4, 6). Based on this, we further evaluated the effect of CLU in NPC metastasis *ex vivo*. 5-8F-shCLU cells were injected into the tail veins of BABL/c mice. In comparison with the mock, the metastasis of 5-8F-shCLU cells to livers, lungs and lymph nodes significantly decreased (Fig. 6B-a, b. *P*<0.05). CLU expression decreased in the metastatic tumors from 5-8F-shCLU cells (Fig. 6B-c, d, e. *P*<0.05), and MMP-9 and VEGF expressions were also decreased in the shCLU group (Fig. 6B-c panel 4, 6). These results suggest that CLU participates in DNP-induced NPC invasion and metastasis *ex vivo*.

## DISCUSSION

In clinic, NPC has a high metastasis clinicopathological features, approximately 95% of NPC cases were undifferentiated carcinomas with high incidence of distant metastases [50, 51]. The metastatic incidence of neck lymph node may be as high as 78.9% [52]. But the reasons of NPC high metastasis are not entirely clear. Multifactorial etiology with dynamic interplay of genetic predisposition, Epstein-Barr virus (EBV) infection and environmental carcinogens is suggested [53]. EBV latent membrane protein-1 and -2 (LMP1 and 2) have been confirmed to promote progression and metastasis of NPC [54, 55], but the positive rate of EBV-LMP expression in NPC is only 61% [56], it don't clearly explain NPC high metastasis.

Recently, chemical carcinogen factors have attracted serious concern [27, 57, 58]. DNP had been thought to be a carcinogen for NPC [16-18, 25]. DNP not only participates in NPC development [21-23], but also promotes NPC metastasis [26, 27, 59]. In experiments concerning DNP-induced rat NPC, DNP showed an organ specificity to nasopharyngeal epithelium and a high metastasis incidence of NPC [26, 60]. In clinical assays, NPC patients with metastasis have a high level of DNP [27]. In this study, DNP treatment increased motility and invasion of 6-10B cell *in vitro*, and promotes 6-10B metastasis *in vivo*. We think that DNP may be one of factors in NPC high metastasis.

CLU highly expresses in various human tumors, and its expression is often correlated with tumor metastasis and poor prognosis [61]. Our clinic study showed that high CLU, MMP-9 and VEGF expressions were significantly correlated with T stage, N stage, and M stage, this suggests that CLU, MMP-9 and VEGF might be important for the acquirement of malignant potential in NPC. Experiment studies showed that CLU expression promoted NPC cell motility and invasion. Animal studies further confirmed that CLU increased NPC cell metastasis *in vivo*. All the above results suggest CLU acts as a pivotal factor contributing to progression of NPC and may be involved in the invasion and metastasis of NPC.

**Figure 7:**
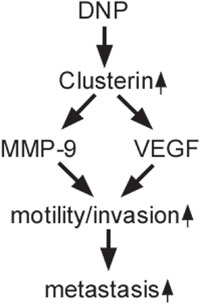
Schematic illustration of DNP-promoted nasopharyngeal carcinoma metastasis through CLU DNP induces CLU expression, binds to and increases MMP-9 and VEGF expression, increases motility and invasion of NPC cancer, and promotes NPC metastasis.

Further, experiment studies showed that DNP could induce CLU, MMP-9 and VEGF expressions, and increase NPC cell motility and invasion through CLU. DNP also induced NPC metastasis in nude mice. MMP-9 are known to remodel the surrounding ECM by proteolysis to invade and metastasize [62]. Recently, MMP-9 upregulation has been documented in several human cancers, including prostate cancer, ovarian cancer, and hepatocellular carcinoma [41, 42, 44, 63]. Additionally, the metastatic process of tumor cells needs to angiogenesis as well as remodeling of the extracellular matrix. VEGF contributing to tumor cell invasion is commonly associated with angiogenesis [47, 48]. In several human cancers, VEGF is highly expressed, where it promotes tumor angiogenesis, providing critical support for tumor growth and survival [38, 39, 64, 65]. Besides, some data also indicated that CLU-induced invasion was through MMP-9 or VEGF [38-40, 49]. In the current study, we demonstrated CLU coexpressed with MMP-9 and VEGF. Increased MMP-9 and VEGF expression, and downregulation of CLU by siRNA or shRNA resulted in the reduction of the MMP-9 and VEGF expression in 6-10B cells induced by DNP. These suggest that DNP may increase MMP-9 and VEGF expression through regulation of CLU, remodeling extracellular matrix (ECM) and promoting tumor angiogenesis, participate in NPC metastasis (Fig. 7).

Together with data from the mechanistic studies, we propose a model in which DNP induces metastasis in NPC cells through activating the MMP-9/VEGF signaling pathway. However, the exact underlying mechanism of DNP action requires further investigation. In addition, we show that CLU, MMP-9 and VEGF can also be detected at the NPC tissues, which could open new avenues for both the tumor metastasis and the development of novel immunotherapeutic strategies in NPC.

## MATERIALS AND METHODS

### Ethics statement

This project's experimental designs and protocols were submitted to the ethical committee at Zhuhai Hospital of Jinan University (Zhuhai, Guangdong, China) before performing the study. The ethical committee members reviewed the experimental designs and protocols to determine whether these studies would hurt the security and privacy of the patients enrolled, and gave ethical approval. Additionally, all patients enrolled agreed to participate in the project and gave signed consent.

### Reagents and antibodies

The chemical structure of DNP was shown at Figure 2A, DNP was supplied by Cancer Research Institute, Central South University (Hunan, China). Antibody against CLU, MMP-9 or VEGF was purchased from Abcam (Cambridge, UK). Antibody against GAPDH and normal mouse IgG were purchased from Upstate Biotechnology, Inc. (Lake Placid, NY). The secondary antibodies, horseradish peroxidase-linked antimouse immunoglobulin G and anti-rabbit immunoglobulin G, were purchased from Santa Cruz Biotechnology, Inc (Santa Cruz, CA, USA). Western-blotting detection regents and BCA protein Assay kit were from Amersham Pharmacia Biotech (Piscataway, NJ). Dimethyl sulfoxide (DMSO), fluorescein isothiocyanate (FITC)-phalloidin, and 4′,6-diamidino-2-phenylindole (DAPI) were purchased from Sigma-Aldrich (St. Louis, MO). DNase I and Rnase were purchased from Qiagen, Inc. (Valencia, CA). Chemical reagents, including Tris, HCl, Sodium dodecyl sulfate (SDS), Na_2_S_2_O_3_, K_3_Fe(CN)_6_, TPCK-Trypsin, NH_4_HCO_3_, acrylamide, urea, thiourea, NP-40, Triton X 100, DL-Dithiothreitol (DTT), phenylmethane-sulfonyl fluoride (PMSF), CHAPS, and pharmolyte were purchased from Sigma-Aldrich. Transwell and Matrigel were obtained from BD Biosciences (Bedford, MA, USA).

### Cell culture and DNP treatment

Human NPC cell lines, 6-10B and 5-8F (sublines derived from cell line SUNE-1) were obtained from Sun Yat-sen University Cancer Center (Guangzhou, China). 6-10B cell line has a low metastatic ability, while 5-8F cell line has a high metastatic ability. The cell lines were cultured in RPMI-1640 medium containing 10% fetal bovine serum (FBS), and were maintained in an incubator with a humidified atmosphere of 95% air and 5% CO2 at 37°C. DNP crystals were dissolved in DMSO, and appropriate amounts of DNP stock solution were added to the culture medium to achieve the indicated concentrations. The cells were then incubated for the indicated times. To investigate the time-dependent of DNP treatment, 6-10B cells were treated with 80 μmol/L DNP for 0, 6, 12, 18 and 24 h. For dose-dependent assays, the cells were treated with 0, 20, 40, 60, 80 μmol/L DNP for 24 h.

### NPC biopsy sample

A total of 175 pathological specimens were collected from January 2013 to June 2015 at First Hospital of Nanhua University (Hengyang, Hunan, China) including 144 cases of primary NPC tissues and 31 cases of normal nasopharyngeal (NNP). All specimens were confirmed by histopathological examination. None of the patients underwent chemotherapy or other adjuvant. 144 patients with NPC were comprised 108 men and 36 women with age from 20 to 71 years (median, 43.6 years). TNM classification was defined according to WHO 2005 NPC staging system [66]. The clinicopathological data of NPC patients are shown in Table 2. 31 cases of NNP included 17 men and 14 women with age range between 17 and 65 years (mean age 43.3 years).

**Table 2:** Primer sequences for quantitative polymerase chain reaction

Gene	Orientation	Primer sequence (5′–3′)
CLU	Forward	TGGCTTCCCACACTTCTGACT
	Reverse	CGCCACGGTCTCCATAAATT
MMP9	Forward	TACCACCTCGAACTTTGACA
	Reverse	AGACAGATCACAGGTACAGG
VEGF	Forward	AGACAGATCACAGGTACAGG
	Reverse	AGCAGGTGAGAGTAAGCGA
18srRNA	Forward	CCTGGATACCGCAGCTAGGA
	Reverse	GCGGCGCAATACGAATGCCCC

### Immunohistochemistry

Immunohistochemistry was done on formalin-fixed and paraffin-embedded tissue sections according to the methods described previously with minor modifications [29]. 5 μm thick tissue sections were deparaffinized in xylene, rehydrated in a graded alcohol series, and treated with an antigen retrieval solution (10 mmol/L sodium citrate buffer, pH 6.0). The sections were incubated with rabbit monoclonal anti-CLU (Abcam, dilution 1:50), mouse monoclonal anti-MMP-9 (Abcam, dilution 1:50), or mouse monoclonal anti-VEGF (Abcam, dilution 1:50) antibody overnight at 4°C. Subsequently, the sections were incubated with a biotinylated econdary antibody (Zhongshan, China), followed by incubation with an avidin–biotin complex (Zhongshan, China) according to the manufacturer's instructions. Finally, the sections were incubated with 3′,3′-diaminobenzidine (DAB) (Sigma-Aldrich) and hydrogen peroxide for 2 min, and counterstained with haematoxylin for 30 s. In negative controls, primary antibodies were omitted.

### Evaluation of staining

The sections were evaluated by two investigators, without prior knowledge of the clinical data, independently graded the staining intensity in all cases. CLU, MMP-9 or VEGF staining was assessed according to the methods described by Zhao [67] with minor modifications. Each case was scored based on the intensity and percentage of cells. At least 10 high-power fields were chosen randomly, and >1000 cells were counted for each section. The intensity of CLU, MMP-9 or VEGF staining was scored as 0 (no signal), 1+(weak), 2(moderate), and 3 (marked). Percentage scores were assigned as 0, 0-25%; 1, 26-50%; 2, 51-75%; and 3, 76-100%. The summed (extension + intensity) was used as the total score. We grouped all samples into the high expression group (total score≥2) and the low one (total score<2) according to the protein expression.

### Enzymatic activity assay

Activities of MMP-9 in the cells and NPC biopsy tissues were assayed using Cell or Tissue Active MMP-9 Fluorescent Assay Kit (Genmed Scientifics Inc. USA) following the manufacturer's instructions. For cell MMP-9 activity assay, the treated cells were washed with phosphate-buffered saline (PBS), and then covered with the washing buffer (supplied in the Kit). After the washing buffer was completely discarded, the cells were harvested with the cell scraping. After being centrifuged, the sediments were treated with the lysis buffer (supplied in the Kit). After the lysates were centrifuged, the supernatant liquids were collected, and used to be measured spectrophotometrically. For tissue MMP-9 activity assay, NPC biopsy tissues were washed with the washing buffer, and then frozen in liquid nitrogen. The frozen tissues were pulverized, and added the lysis buffer. After the lysates were centrifuged, the supernatant liquids were collected, and used to be measured MMP-9 activity. The relative fluorescence units were determined with an excitation wavelength of 330 nm and an emission wavelength of 400 nm. The consistency of fluorescent polypeptide segmengts was calculated on the basis of the relative fluorescence units, MMP-9 activities were expressed as nmol/mg/min.

### CLU, MMP-9, VEGF gene expression analysis using RT-qPCR

Reverse transcription quantitative polymerase chain reaction (RT-qPCR) with a SYBR® Green reporter was used to detect *Clu*, *Mmp-9*, *Vegf* gene mRNA. Briefly, total RNA was isolated using Trizol (Invitrogen Co., Shanghai, China) reagent following the manufacturer's instructions. The RNA samples were reverse-transcribed to cDNA using a PrimeScript® RT Master Mix kit (Takara Co., Dalian, China). Gene-specific primers were combined with SYBR® Premix Ex Taq™ (Takara) and amplified using an ABI 7500 Real-Time PCR System (Applied Biosystems, Foster City, CA, USA). All qPCR reactions were conducted independently on five samples. The relative mRNA expression levels were calculated using the 2^−ΔΔCt^ method. Primers for *Clu*, *Mmp-9*, *Vegf* genes and 18srRNA were supplied by Qiagen. The primer sequences are described in Table 2.

### Construction of expression vectors

pcDNA3.1 and pSR-GFP/Neo-shRNA vectors were purchased from Invitrogene (Invitrogene Co., CA). *Clu* DNA fragment was generated by PCR, and cloned into Bam H I/Xho I of the pcDNA3.1 vector (Amersham Biosciences Corp., Piscataway, NJ) to generate pcDNA3.1-CLU plasmids. The primers were synthesized for the CLU-BamHIF (*5′-CGCGGATCCGCCACCATGATGAAGACTCTGCTGCTGTT-3*′) and for the CLUXhoIR (*5′-CCGCTCGAGTCACTCCTCCCGGTGCTTTTTGC-3′*). The pSR-GFP /Neo-shRNA vector was used to construct pSR-GFP/Neo-NC-shRNA (shmock) and pSR- GFP/Neo-CLU-shRNA (shCLU) following the recommended protocol. The primers were synthesized for the shmock (general scramble: sense *5′- GATCCTTCTCCGAACGTGTCACG TTTCAAGAGACGTGACACGTTCGGAGAATTTTTTTTT G-3*′, anti-sense *5′- AATTCAAAAAAAAATTCTCCGAAC GTGTCACGTCTCTTGAAACGTGACACGTTCGGAGAA G-3*′), and for shCLU(general scramble: sense *5′-GAT CCGGATGAAGGACCAGTGTGACAAGAGTCACA CTG GTCCTTCATCCTTTTTTTG-3*′, antisense *5′-AATTCAA AAAAAGGATGAAGGACCAG TGTGACTCTTGTCACAC TGGTCCTTCATCCG-3′*. All constructs were confirmed by restriction enzyme mapping, DNA sequencing and blast.

### siRNA and transfection

*Clu* siRNA (siCLU) sequences synthesized by Sigma-aldrich (St. Louis, MO) were as follows: sense *5′-GGAUGAAGGACCAGUGUGAdTdT-3*′, antisense *5′-UCACACUGGUCCU UCAUCCdTdT-3*′. When growing 40% confluence, 6-10B cells were transfected with siCLU using Lipofectamine 2000 reagent (Life Technologies, Inc.) following the manufacturer's suggested protocol. In parallel, cells transfected with nonspecific siRNA (simock) (sense *5′-UUCUCCGAACGUGUCACGUTT-3′,* antisense *5′-ACGUGACACGUUCGGAGAATT-3*′) were used as control. simock was also purchased from Sigma-Aldrich. pSR-GFP/Neo-NC -shRNA and pSR- GFP/Neo-CLU-shRNA were transfected in to 5-8F cells with Lipofectamine 2000. shCLU-5-8F and mock stably-transfected cell lines were obtained by selection for G418 resistance (400 mg/ml) and further confirmed by assessing CLU expression. The stably-express cell lines were then subjected to Western-blotting analysis, invasion, and motility assay.

### Cell invasion and motility assay

Boden chamber invasion assay was performed with minor modifications as described previously by Tang et al. [27]. For invasion assay, the treated cells were removed by trypsinization, and their invasiveness was tested using Boyden chamber invasion assay *in vitro*. Matrigel (BD, Bedford, MA) at 25 mg/50 mL was applied to 8-mm pore size polycarbonate membrane filters. The cells were seeded into the upper part of Boyden chamber at a density of 1.5 × 10^4^ cells/well in 50 μl of serum-free medium, and then incubated for 12 h at 37°C. The bottom chamber also contained standard medium with 20% FBS. The cells invaded to the lower surface of chamber membrane were fixed with methanol and stained with crystal violet. The number of invaded cells per field was captured using a microscope fitted with a camera at 200×magnification. The motility assay was carried out as described in the invasion assay with no coating of Matrigel.

### Immunoprecipitation assay

Immunoprecipitation assay was used to analysize CLU interaction with MMP-9 or VEGF previously described [26]. The treated cells were washed once with ice-cold PBS, then harvested and disrupted in lysis buffer [25 mM Tris-HCl (pH 7.5), 5mM β-glycerophosphate, 0.1 mM Na3 VO4, 10 mM MgCl2, 1 mM aprotinin and 1 mM PMSF]. The lysates were centrifuged at 12,000 rpm for 30 min at 4°C. The concentration of supernatant fraction was measured using BCA protein Assay kit. 100μg (100μl) supernatant fractions were added 20μl glutathione-Sepharose 4B with CLU antibody, incubated for overnight at 4°C. After being washed 3 times, the glutathione-Sepharose 4B with immunocomplexes was collected. The immunocomplexes were subjected to Western-blotting analysis.

### Western-blotting

Western-blotting analysis was performed as previously described [28]. The cell lysates were prepared, and protein concentrations were measured by BCA protein Assay kit. 40μg cell lysates were denatured in 5 × sample loading buffer by heating at 95°C for 10 min. The denatured samples were then separated by 10% polyacrylamide gel. The separated proteins were transferred onto a nitrocellulose membrane (Bio-rad). The membranes were subsequently incubated with 5% non-fat milk in Tris-buffered saline containing 0.05% Tween-20 (TBST) for 1 h to block non-specific binding. The treated membranes were incubated with antibody against CLU, MMP-9, VEGF or GAPDH overnight at 4°C. After being washed, the membranes were incubated with the secondary antibody for 1 h at room temperature. Finally, target proteins were detected by ECL (Pierce, Rockford, USA). GAPDH was used as an internal control to verify basal level expression and equal protein loading. The abundance ratio to GAPDH was counted.

### Xenograft model

20 female BALB/c nude mice (approximately four to six weeks old) were obtained from Animal Center of Central South University, and quarantined for one week prior to tumor implantation. 100 μl 6-10B or 5-8F cell suspensions (1 × 10^4^ cells) were mixed with Matrigel, and then injected into the tail veins of nude mice (10 mice per group) respectively. In the DNP group, the nude mice were injected subcutaneously with DNP at a dosage of 40 mg/kg (bodyweight), twice a week for 60 days [26]. The nude mice were scarified, metastases at liver, lung and mediastinal lymph nodes were evaluated by measuring weight of the metastasized tumors on 60^th^ day. The metastatic tumor tissues were collected for Western-blotting analysis. Animal welfare and experimental procedures were followed strictly. This study was approved by the ethics committee of Jinan University.

### Statistical analysis

Data analysis was performed using SPSS version 17.0 software (SPSS, Inc., Chicago, IL, USA). The results were expressed as the mean ± standard error of the mean from a minimum of three independent experiments. The statistical significance between groups was determined by one-way analysis of variance. *P*<0.05 was considered to indicate a statistically significant difference.

## SUPPLEMENTARY FIGURES


