# Supplementary material for: Clusterin induced by N,N′-Dinitrosopiperazine is involved in nasopharyngeal carcinoma metastasis
Source: Oncotarget. 2015 Dec 24;7(5):5548–63. doi: 10.18632/oncotarget.6750 (PMC4868705; doi:10.18632/oncotarget.6750)
Supplement: Supplementary file 1 [file oncotarget-07-5548-s001.pdf]

SUPPLEMENTARY FIGURES

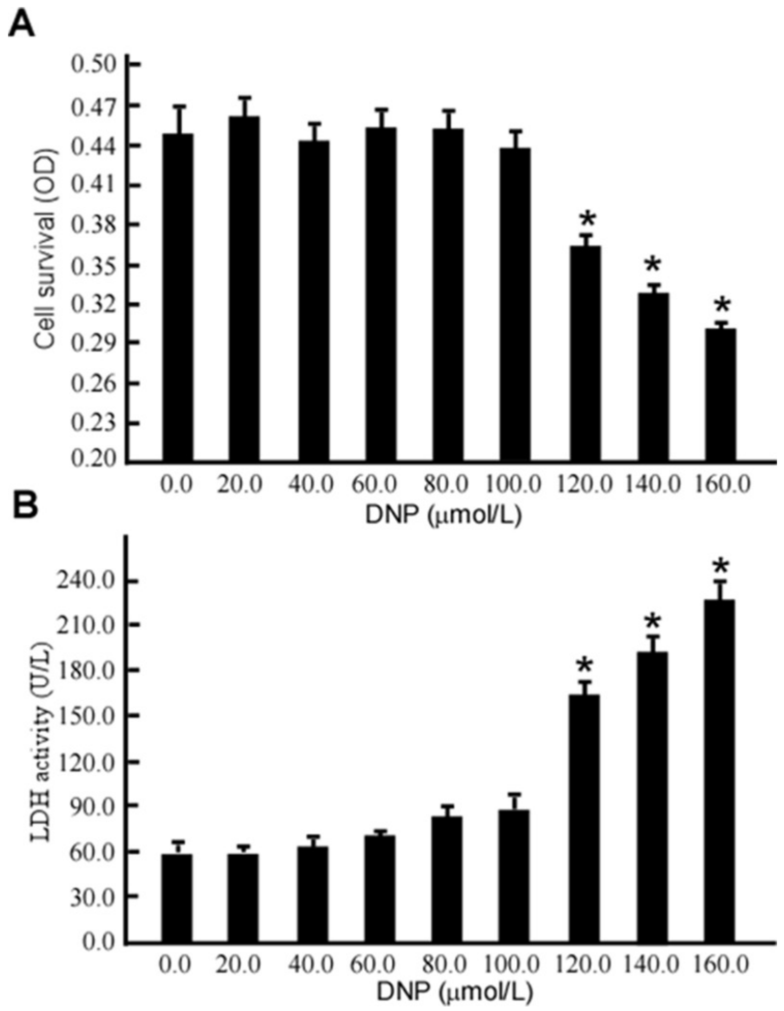

Supplementary Figure S1: Assay of non-cytotoxic concentrations (NCC) of DNP to 6-10B cells. A. MTT assay was used to determine the NCC of DNP to 6-10B cells. Briefly, 5×10^3 6-10B cells were seeded in 96-well plates, and treated with DNP at 0 to 160 μmol/L for 24 h. 20 μl MTT (Sigma-Aldrich) at 0.5% was added to the cell wells for 4 h. The viable cell number was directly proportional to the production of formazan, which was solubilized in isopropanol and measured at 492 nm. **B.** LDH assay was used to confirm the NCC of DNP. Briefly, 2×10^4 6-10B cells were seeded in 6-well plates, and treated with DNP at 0 and 160 μmol/L for 24 h. The media were collected for LDH activity measurement using the LDH assay kit (Autec Diagnostica). LDH, lactate dehydrogenase. MTT, 3-(4,5-dimethylthiazol-2-yl)-5-(3- carboxymethoxyphenyl)-2-(4-ulfophenyl)-2H-tetrazolium. *p< 0.05.

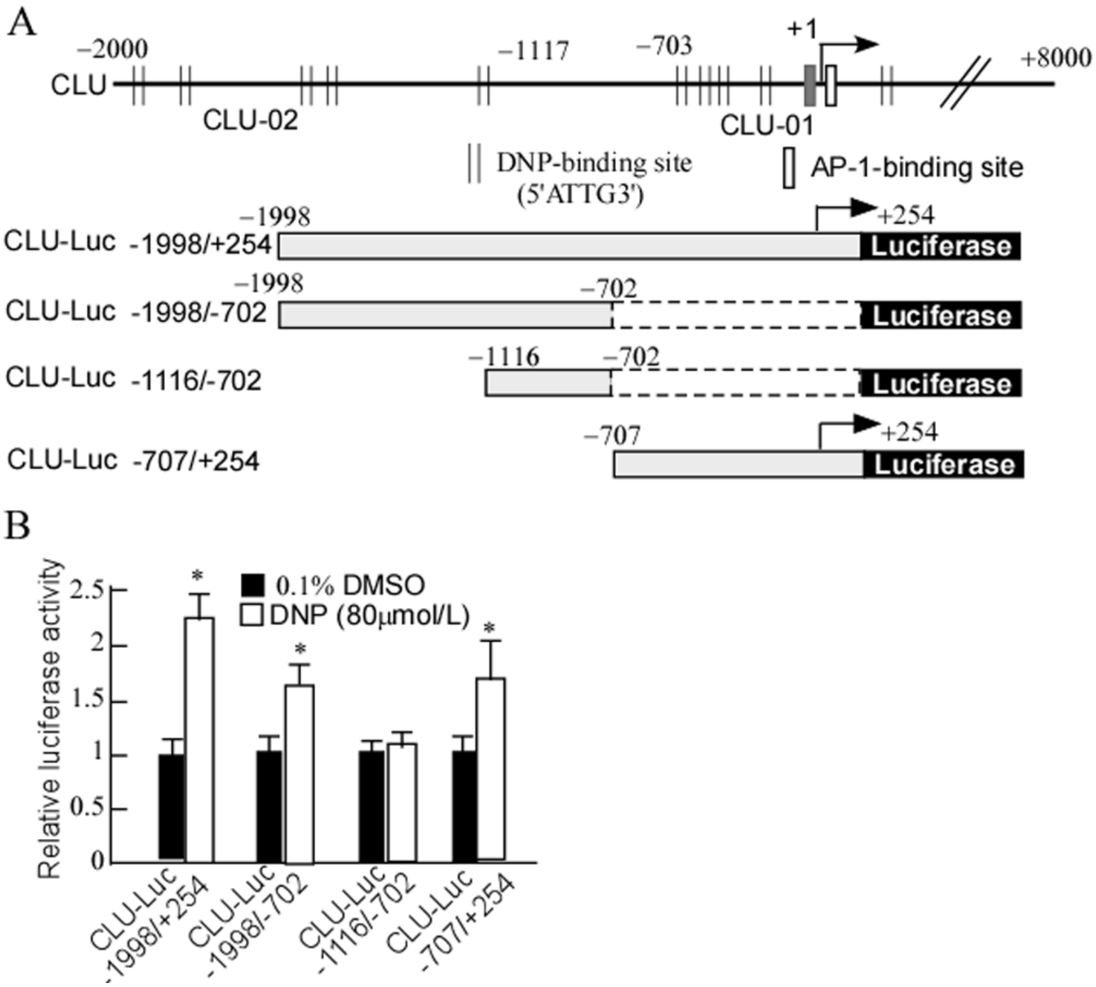

Supplementary Figure S2: The binding sites of DNP with CLU promoter. A. The putative DNP binding sites were calculated in the CLU promoter region between- 2000 bp and +500 bp from transcription start site using computer model. Ten binding sites were identified in CLU promoter regions. Various lengths of the promoter and partial first exon of the wild-type CLU gene were amplified by PCR using genomic DNA and the following primer pairs: P1:*5'CTCGAGCATGGCAGGTAGTGAGCTCCCTG3'*; P2:*5'A GATCTGTGTCCAGAGGGGTTTGCT3'* for CLU–Luc -1998/-702. P3:*5'AGATCTGATTTCCTAACTGGGAAGG3'* and P4:*5'AAGCT TGAGCTGTGTCATCCCTCTCTGCCT3'* for CLU– Luc-707/+254. PCR products were cloned into pGL3-vector (Promega). CLU–Luc-1998/ +254 was constructed by inserting CLU–Luc-707/+254 fragment. CLU–Luc-1116 /-702 was constructed from CLU–Luc-1198 /-702 by deletion of SmaI and PshAI fragment. **B.** 6-10B cells were transfected with the indicated reporter plasmid, pRL-TK transfect as an internal control. After DNP treatment, luciferase activities were measured using Dual-Luciferase Reporter Assay System (Promega) and a microplate luminometer (EG&G Berthold). The firefly luciferase activities were corrected by corresponding Renilla luciferase activities. The results are repeated at least 3 experiments.

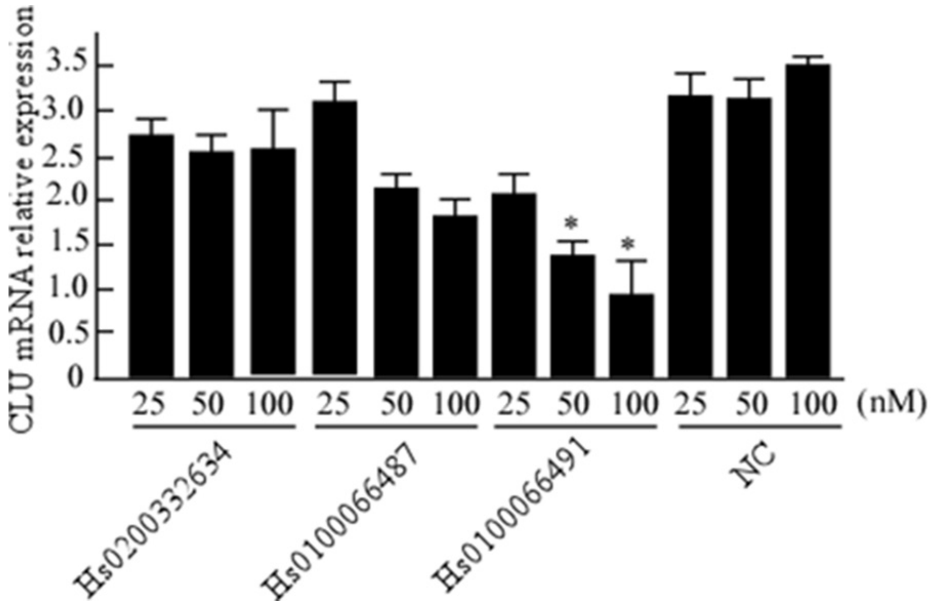

Supplementary Figure S3: The inhibitory effect of shCLUs on CLU. Three shCLUs were designed and synthetized by Sigma Company (Shanghai, CN), HS0100066487 (5'*GAAAGAGGAUGCCCUAAAUdTdT*3'; 5'*AUUUAGGG CAUCCUCUUUCdTdT*3'), HS0200332634 (5'*GAAAUACAACGAGCUGCUAdTdT*3'; 5'*UA GCAGCUCGUUGUAUU UCdTdT*3') and HS0100066491 (5'*GGAUGAAGGACCAGUGUGAdTdT*3'; 5'*UCACACUGG UCCUUCAUCCdTdT*3'). NC (5'*UUCUCCGAACGUGUCACGU TT*3'; 5'*ACGUGACACGU UCGGAGAATT*3') is the control. 5×10^4 5-8F cells were transiently transfected with these shCLUs at 25, 50 100nM, respectively. After 24 h, RNAs in the transfected cells were extracted using Trizol(Invitrogen)following the instruction. CLU mRNA was detected in the RNA samples using conventional Real-time PCR with the specific primers. The inhibitory rates of three shRNAs were calculated using the formula [(NC 2^{-CT}-siRNA 2^{-CT})/NC 2^{-CT}100%]. *$p < 0.05$.
